# Gold Can Be so Manipulated as to Make Operations Permanent

**Published:** 1878-10

**Authors:** Marshall H. Webb


### ARTICLE III.

### *Gold can be so Manipulated as to make Operations Permanent.*

BY MARSHALL H. WEBB.

Chalk is carbonate of lime, and there is none of this in any dental tissue; therefore, no tooth can be compared, in any way whatever, to "a little block of chalk." In the *Dental Miscellany* for April, 1878, Dr. C. M. Wright, of Basil, Switzerland, has attempted to make such a comparison, but he has only again demonstrated that he is not fine enough in his perceptions (and cannot, therefore, be accurate enough in his manipulations) to be a good judge and a thorough and skillful operator.

When operations have been so performed as to entirely prevent fluids from entering between the gold and the tissue or tissues, against which it has been built, there is certainly the same action going on in the protoplasmic elements between the enamel prisms, as before, but this process is a fulfillment of a law in the animal economy, and is not to be compared to the simple infiltration of fluid through the wall of a cylinder of non-living vegetable tissue (such as Dr. Wrights' "rattan model" and to the gold which may be placed in it.

Dr. Wright asks, "How long should a filling remain to be considered permanent?" If gold (or any other material) is used as a simple "stopping" it will, in all probability, be only "temporary," but in the hands of properly qualified and skillful operators, it can be so manipulated as to make operations permanent. Permanent signifies "continuing in the same state, or without any change that destroys form or character; remaining unaltered or unremoved; continuance in the same state or place; duration; fixedness." So far as the materials used in the performance of dental operations are concerned, therefore, gold is the only material that can be made to answer all these requirements. Operations can be so performed as to prevent further disintegration of enamel about the parts against which gold has been well impacted and finely finished. Gold can be so manipulated as to aid in the preservation of the teeth so long as the organ or organs operated upon are not attacked by caries in other parts, and until solution and absorption of the alveolar process takes place, and the teeth loosen and fall out, or till the death of the patient, and the remanding back of the elements of the tissues to their primal condition.

We are having no "earthquake," such as Dr. Wright refers to, in America, but there are practitioners who are *Flagging*, and attributing the cause of failure in dental operations to gold as being incompatible with tissue, when in reality the principal cause of failure consists in their incompatibility with their calling.

In such cases as Dr. Wright refers to as coming in for treatment in Switzerland—in very many cases everywhere—function has been interfered with or interrupted, or nutriment has not been sufficiently rich and abundant, and tissues have not been perfected, and it is in just such cases that the most thorough and careful treatment and perfect operations are required. If an operator does not possess the ability to properly perform an operation with gold, it were better for him to insert another material than to extract or cause the loss of the organ. In teeth, where calcification has not fully taken place, and where the interglobular spaces, filled with larger protoplasmic elements, might yet be found, it is of the utmost importance to so perform operations, when disintegration penetrates the enamel upon a proximate surface of a tooth, as to have the gold built out to the original contour of the missing tissue, and to come closely in contact with that which may be inserted in, or with the normal tissue of, the adjoining tooth. Each such operation should be so performed that the enamel, against which the gold has been built and finely finished, shall be free from contact at every part, and be washed by the saliva.

With proper hygienic management, the tissues of such organs as have just been referred to, will gradually but surely become more fully calcified and perfected, year by year, so that at maturity the acid conditions, concomitant with active carious processes, will be no longer present, to the same extent, and the teeth be of that character not liable to be attacked by destructive agents. Each operator should do all that he possibly can to preserve the pulp in every case presented, and especially in all cases where the tissues of the body have not matured. It is even better to retain a pulp that is in a semi-vital condition than to destroy it while the patient is yet in his or her teens, because, in youth the balance is in favor of supply of pabulum, not only to sustain but to perfect tissues.

It is not only necessary for one to possess the genius of an artist to become a first-class operator, but he must also be an anatomist and histologist; a physiologist and pathologist; an etiologist and a therapeutist, and be able to intelligently apply remedial agents for the "removal of abnormal functions and abnormal growths, that the physiological energies may hold normal sway in the production and maintenance of the elements of the tissues and organs." It is not of so much importance to "employ gold, amalgam, tin, oxychloride of zinc, gutta percha and judgment," and to "use gold in cylinders, pellets, strips, balls, ribbons, mats, ropes, blocks," &c., &c. (none of which forms are the best to use,) or to "employ automic and engine mallets, and pretty Swiss girls as vital mallets (?.) hand-pressure and all other well-known methods" (excepting the very best;) it is far better to consider *how* all these things can best be employed—how operations can best be performed by their aid. There are very many practitioners in almost every part of the world who have "modern charts, stools, dam-appliances, engines, instruments, &c.," and each might be "an earnest and enthusiastic *dentist*," and yet by no means a capable and skillful operator.

Several years ago a thoroughly educated, honest and conscientious practitioner, and first-class operator, was called to give a clinic before the Iowa State Dental Society. He took with him the instruments which he usually employed to perform operations, and after giving a clinic he learned that a practitioner, who was present, had said that it was "easy enough for Dr. Atkinson to perform such an operation with such fine instruments," whereupon the same practitioner was invited to witness a clinic to be given the following day by the same operator. When the time arrived for the performance of the second operation, a simple file was made into an excavator (by the operator) and the cavity was prepared; when this was done, the same point was made into one of a proper shape for inserting the gold, which was all impacted by the aid of this same crude instrument, and a plain hand-mallet or hammer. After this was done, the same end of the file was made into a chisel and, by this means, together with the file-cut surface of the file, the surface of the gold was smoothly finished, and the operation was equal or even superior to the one performed the day previous by the aid of fine instruments. In this case, the success of the operation depended upon the genius of the operator, and not upon the use of appliances.

Dr. Wright states that the "filling of teeth is a very absorbing occupation, but it is not a *fine art*." An operation can be made a work of "*fine art*" if the operator be really an artist. "Our monuments" *do* "express the nobler sentiments of human mind" if the operator has the ability to "express" and the mind have the "nobler sentiments."

Most of the patients of a thoroughly conscientious, capable and skillful operator "really appreciate the work we do" more than the majority of "our craft," because such majority do not know what fine operations are—what "*fine art*" is. Could we but "see ourselves as" this same majority of so-called dentists have led "others" to "see us," there are but comparatively few who would have any enthusiasm, without which key-note real success cannot be attained. While it may require an encouraging word (as in the case of Michael Angelo with his Madonna,) to kindle that inspiration which leads to the production of a work of art, yet all artists obey the behests of genius without taking into the account the mere opinions of others. When we are governed by the "nobler sentiments of the human mind," as other artists are, and do all in our power to perform first-class operations, and make each and every operation a specimen of "*fine art,*" then will we deserve and have "brotherhood with the painters and sculptors" (which latter we ought to be,) and also to be the "architects" not only of fine work but of our own "fame" and that of our calling.—*Dental Miscellany.*

